# On the Diseases of the Interior Valley

**Published:** 1854-12

**Authors:** 


ART. VIII.—*A Systematic Treatise, Historical, Etiological, and Practical, of the Principal Diseases of the Interior Valley of North America, as they appear in the Caucasian, African, Indian, and Esquimaux Varieties of its Population.* By DANIEL DRAKE, M. D. Edited by S. HANBURY SMITH, M. D., formerly Prof. of Theory and Practice in Starling Medical College, Ohio, and FRANCIS S. SMITH, M. D., Prof. of Institutes of Medicine in the Medical Department of the University of Pennsylvania College. Second Series. Philadelphia: Lippincott, Grambo & C., Publishers. 1854.

The title-page of this book indicates its comprehensive character. There was no little heroism in the mere project of such a work. To undertake it, one should have large scientific attainment, vigorous powers of generalization, health, strength, youth, money, and a never-failing self-reliance. All of these attributes, save youth, and health, and money, were centered in Dr. Drake; and his work, gigantic in design, though incomplete in execution, will stand as the noblest and most fitting monument of the man and the mind that conceived it.

Sepulchral architecture sometimes erects a broken shaft over the grave of the young and gifted, indicative of early hope and youthful promise, shattered and destroyed. No broken shaft should shadow the ashes of Drake. The solid foundation, the broad imperishable plinth, the tall and stately column, all are there—the capital alone is wanting. The coronal of oak and laurel might wreath about its sides indicative of triumphs already achieved, but the richly carved and decorated crown, the finishing stroke of emblematic art, remains unwon—for life, alas! is short!

Drake had laid out for himself a labor to which one human life is insufficient. Patiently and laboriously he had toiled along, accomplishing results which were truly wonderful; but while he slowly gathered the rich harvest of the wide field he traversed, the reaper Death removed him to the universal garner; and other hands have winnowed his grain.

What Drake calls the "Interior Valley," is the whole of that broad and fertile region which is drained by the Mississippi. Its diseases are at once peculiar and widely variant. The Mississippi river alone crosses twenty-one degrees of latitude. At Baton Rouge, in the 30th parallel, the average temperature of the year is 67° 56′. At Fort Snelling, on the Upper Mississippi, latitude 44° 53′, the average temperature is 44° 57′. The district thus presents a difference of 23° in the yearly mean of temperature.

Accompanying this variance of heat are other circumstances equally important. The clear running streams and rocky hills of the mountain declivities are contrasted with the stagnant bayou and muddy lake of the alluvial region. In one part a dry and sandy soil nourishes a sparse vegetation, while in another, a luxuriant forest growth is rapidly produced, destroyed, and reproduced. Here an arid plain extending across successive degrees of latitude and longitude, without streams or lakes, offers no sources of humidity; while in another, the presence of sluggish rivers, of frequent floods, and swampy bottom lands, impregnates the air with moisture and develops the decomposition of organic matter.

In analyzing the causes of disease thus presented, the mind of Drake has astutely fixed upon the universal presence of moisture in unhealthy localities. Vegetable decomposition, forests, and other sources of miasma, may exist without harm until the addition of the humid element develops their noxious qualities and gives them power for evil. "Water is a necessary element in all the hypotheses which have been framed to account for autumnal fever."

In reference to heat as a cause of disease, he reasons that although the autumnal fever prevails perpetually, and virulently within the tropics, while it entirely ceases before we reach the polar circle, it is evident that heat alone is not the active agent. For, as he instances, though the people of Mobile suffer intensely, the inhabitants of the adjoining oak and pine terrace enjoy an immunity from the diseases which depopulate the neighboring town. A thousand such cases testify to the fact that heat alone is an exciting, and *not* a predisposing cause.

Living vegetation has, in Dr. Drake's opinion, no assignable share in the cause or prevention of autumnal fevers. To decaying vegetable matter and to tellurial gases, generally, he accords an important and essential part, and speaks of the former as a necessary condition to the production of fever, (autumnal).

At the time when Dr. Drake ceased from his labors, no sufficiently extended or accurate observations had been made to determine the agency of humidity alone in the production of disease. His acute mind had not failed, however, to recognize in a high dew-point a condition which would harmonise many conflicting theories, and account for many hitherto unexplained phenomena.

So far as our own observation has extended, we are inclined to assign to humidity alone, unattended by what are meant in the word miasmata, an important share in the causation of epidemics.

The existence of miasma is only known by inference. It has never been proved. The two conditions most changeable, most appreciable, and most likely to affect the functions of the animal system, are heat and humidity. The latter is, to a certain extent, implied in the former, as a heated atmosphere always contains more moisture than a cold one.

No one can read this last volume of Dr. Drake's, without finding in it the best, because unconscious, evidence of the action of humidity in disease.

We regret that our limits do not allow us to pursue further a subject so kindred to our habits of thought, and our pursuits. We can only wish to this last monument of Drake's genius an extended circulation, and a prosperous result to those to whose benefit it will enure. It will assume the importance of a standard work in every library where it has a place.

For sale by PHINNEY & Co. H.

